# Early Neurodevelopment of Extremely Preterm Infants Administered Autologous Cord Blood Cell Therapy: Secondary Analysis of a Nonrandomized Clinical Trial

**DOI:** 10.1001/jamanetworkopen.2025.21158

**Published:** 2025-07-03

**Authors:** Lindsay Zhou, Abdul Razak, Courtney A. McDonald, Tamara Yawno, David T. McHugh, Gillian Whiteley, Kristyn Connelly, Vathana Sackett, Suzanne L. Miller, Graham Jenkin, Iona Novak, Rod W. Hunt, Atul Malhotra

**Affiliations:** 1 Monash Newborn, Monash Children’s Hospital, Melbourne, Australia; 2 Department of Paediatrics, Monash University, Melbourne, Australia; 3 The Ritchie Centre, Hudson Institute of Medical Research, Melbourne, Australia; 4 Department of Obstetrics and Gynaecology, Monash University, Melbourne, Australia; 5 Diagnostic Imaging, Monash Children’s Hospital, Melbourne, Australia; 6 Allied Health, Monash Children’s Hospital, Melbourne, Australia; 7 Cerebral Palsy Alliance, Sydney, Australia; 8 Faculty of Medicine and Health, University of Sydney, Sydney, Australia

## Abstract

**Importance:**

Umbilical cord blood–derived cells (UCBCs) are increasingly being evaluated for neuroprotective properties in perinatal brain injury.

**Objective:**

To report early neurodevelopmental outcomes of extremely preterm infants who received autologous UCBCs in the CORD-SaFe study.

**Design, Setting, and Participants:**

This study reports early follow-up on the preplanned secondary aims of a phase 1 safety and feasibility nonrandomized clinical trial conducted between May 2021 and November 2023, with early follow-up completed in August 2024. Participants were infants born at less than 28 weeks’ completed gestation who received autologous UCBCs in the CORD-SaFe study at Monash Children’s Hospital, Australia. A contemporaneous cohort of noninfused infants born during the study period was included for comparison. Data were analyzed from October to December 2024.

**Intervention:**

Autologous UCBC administered intravenously in the second postnatal week of life.

**Main Outcomes and Measures:**

Infants underwent brain magnetic resonance imaging to assess structure and injury (Kidokoro score) at term-equivalent age. Assessments at 52 to 54 weeks postmenstrual age included General Movements Assessment, Hammersmith Infant Neurological Examination score, and clinical examination to diagnose risk of cerebral palsy.

**Results:**

A total of 23 infants (median [IQR] gestation, 26 [25-27] weeks; median [IQR] birth weight, 748 [645-981] grams; 17 [73.9%] male) were administered UCBCs at a median (IQR) dose of 42.3 (31.1-63.2) million cells/kg. The contemporaneous cohort included 93 infants (median [IQR] gestation, 26 (24-27) weeks; median [IQR] birth weight, 769 [660-1017] grams; 39 [41.9%] male). Median (IQR) Kidokoro score was 2 (1-3) for the UCBCs group and 3 (2-5) for the contemporaneous cohort, with no statistically significant difference observed between the groups (adjusted median difference, 0 [95% CI, −1.78 to 1.78]). No infants in the UCBC group were assessed as high risk for cerebral palsy compared with 6 of 87 assessed infants (6.8%) in the contemporaneous group; however, the difference was not statistically significant (adjusted log odds, 0.31 [95% CI, −0.76 to 1.38]). No differences in Hammersmith Infant Neurological Examination score (adjusted log odds, −1.50 [95% CI, −5.78 to 2.78]) and absent fidgety movements (adjusted odds ratio, 0.24 [95% CI, 0.20 to 3.04]) were observed between groups.

**Conclusions and Relevance:**

This phase 1 nonrandomized clinical trial assessing the safety and feasibility of autologous UCBCs in extremely preterm infants did not find significant differences in brain imaging parameters and early neurodevelopmental outcomes between the cell therapy and contemporaneous untreated groups. It was encouraging to note no infants who received UCBCs were assessed as high risk for cerebral palsy at 52 to 54 weeks postmenstrual age, and the absence of high risk for CP merits further study.

**Trial Registration:**

ANZCTR.org.au Identifier: ACTRN12619001637134

## Key Points

**Question:**

What were term equivalent–age brain magnetic resonance imaging findings and early neurodevelopmental outcomes at postmenstrual age 52 to 54 weeks for infants born extremely preterm who received autologous cord blood cell therapy in the CORD-SaFe study?

**Findings:**

In this secondary analysis of a nonrandomized clinical trial of 23 infants who received autologous cord blood cell therapy compared with 93 noninfused infants, there were no significant differences in brain imaging scores, movement and neurological measures, and early neurodevelopment outcomes. No infants in the CORD-SaFe study received an early diagnosis of cerebral palsy.

**Meaning:**

These outcomes were reassuring for extremely preterm infants who received autologous cord blood–derived cell therapy, and the absence of high risk for CP merits further study.

## Teaser

This preplanned secondary analysis of a nonrandomized clinical trial describes early neurodevelopmental outcomes of extremely preterm infants who received autologous umbilical cord blood–derived cells in the nonrandomized CORD-SaFe study.

## Introduction

Despite advances in neonatal intensive care and overall survival, brain injury continues to be a significant cause of morbidity and mortality in preterm infants, especially in those born extremely preterm (<28 weeks’ completed gestation).^1^ In a recent study from Monash Children’s Hospital in Melbourne, Australia, rates of severe intraventricular hemorrhage and significant white matter injury, such as periventricular hemorrhagic infarction and periventricular leukomalacia, have been increasing over the last decade in very preterm infants.^2^ These brain injuries can lead to death in the neonatal period and to neurodevelopmental impairments in survivors. Physical impairments, especially in infants with early features of cerebral palsy, can now be diagnosed in early infancy (corrected age 3-4 months), while other developmental and cognitive impairments are diagnosed at corrected age 2 years.^3,4^

Limited therapies exist to prevent or treat preterm brain injury, and it is imperative to trial new interventions.^5^ For many years, umbilical cord blood has been used for bone marrow transplantation in the context of hematological malignant neoplasms and disorders, but umbilical cord blood–derived cell (UCBC) therapy has gained much interest over the last few years for neuroprotective properties, including anti-inflammatory, immunomodulatory functions in preclinical models of preterm and perinatal brain injury.^6,7^ Early-phase clinical trials of UCBC therapies have also been completed or are ongoing, with promising results. However, most of these studies have focused on term infants or preterm infants born at more than 28 weeks’ gestation.

As extremely preterm infants are the group most vulnerable and at highest risk of brain injury and subsequent neurodevelopment impairment, we explored the potential of using the infant’s own UCBCs, autologous UCBCs, for preterm neuroprotection during the period where brain injury is most prevalent. We recently reported on the primary aims of the phase 1 trial, the CORD-SaFe study, which examined the feasibility and safety of autologous UCBC administration in extremely preterm infants.^8^ The aim of this study was to report on secondary aims of the CORD-SaFe study: term-equivalent age brain magnetic resonance imaging (MRI) findings and early neurodevelopmental outcomes of extremely preterm infants who received autologous UCBC administration, and compare them to a contemporaneous cohort of comparable preterm infants who did not receive UCBC therapy

## Methods

The CORD-SaFe nonrandomized clinical trial was approved by the Monash Health Human Research Ethics Committee and registered with the Australian New Zealand Clinical Trials Registry. Informed, written antenatal consent was obtained for participants in the intervention group. For the contemporaneous cohort included in this preplanned secondary analysis, the ethics committee approved collection of clinical data and follow-up assessments without informed consent because they were conducted as part of routine clinical practice at the study institution. This study is reported according to the Transparent Reporting of Evaluations With Nonrandomized Designs (TREND) reporting guideline for nonrandomized clinical trials.

Detailed protocol and methods for the CORD-SaFe study (including cord blood collection, preparation of UCBCs, infusion and monitoring) have been published elsewhere.^8,9^ The trial protocol for this study is provided in Supplement 1. In brief, this was a single-center, prospective, phase 1 open-label safety and feasibility clinical trial conducted at Monash Children’s Hospital, Melbourne, Australia, with infants enrolled between May 2021 and November 2023. 

### Participants

Extremely preterm infants born before 28 weeks’ completed gestation (and surviving at 2 weeks of postnatal life) whose parents had consented to participation and who had adequate good manufacturing practice (GMP) grade autologous UCBCs available for intravenous infusion between postnatal days 9 and 15 were included. Infants were excluded if they were outborn, less than 7 mL of cord blood was collected, GMP grade UCBCs were not available, severe intraventricular hemorrhage (IVH) was reported on day 8 cranial ultrasonography, or they had culture-positive sepsis within 48 hours of planned UCBC infusion. There were no changes made to inclusion or exclusion criteria for participants after commencement of the trial.

### Intervention Group

If the infant met inclusion criteria for UCBC infusion between postnatal days 9 and 15, the autologous cryopreserved cord-blood unit was transferred from a Therapeutic Goods Administration–approved cord blood processing facility (Cell Care Australia) in a cryo-shipper to the GMP-adherent Cell Therapies Platform at the Hudson Institute of Medical Research, Melbourne, Australia, for thawing and preparation for UCBC infusion. On the day of infusion, the unit was thawed, twice washed with 10% dextran and 4% human albumin, and UCBCs were resuspended in 4% albumin and 10% dextran at an intended dose of 25 to 50 million UCBCs per kilogram of body weight. The final volume was made up to 10 mL/kg and passed through a 200-µm blood product filter prior to dispensing to the neonatal unit for UCBC infusion. The autologous UCBC infusion was administered intravenously via a 24G intravenous cannula, using a 20-mL syringe, vertical syringe pump, and 75-cm plastic line with no additional filter, over 60 minutes.

### Contemporaneous Cohort

A contemporaneous cohort of similar infants (nonrandomized) were included, as prespecified in the protocol for comparative purposes, and the data were collected prospectively. This cohort included all infants born at less than 28 weeks’ completed gestational age at Monash Medical Centre during the study period but not enrolled in the trial. The same exclusions were applied as for the UCBC infusion group: outborn, severe IVH on D8 cranial ultrasonography, and survived beyond postnatal week 2.

### Outcomes

For this study, we included data on early neurodevelopmental outcomes for both the intervention and contemporaneous cohort. Term equivalent–age MRI brain scans (all conducted after cell infusions in the cell therapy cohort and performed using standard acquisition protocols on a 3-Tesla MRI machine) were evaluated for preterm MRI brain injury scores (Kidokoro) and measurements of important brain structures on cross sections of cerebrum, cerebellum, and brain stem imaging planes by independent researchers who were not aware of the group allocations.^10,11^ The Kidokoro score assesses cerebral white matter, cortical gray matter, deep gray matter, and cerebellum, combining to form a global brain injury score described as normal (0-3), mild (4-7), moderate (8-11), or severe (≥12).^10,11^ Early neurodevelopmental assessments included General Movements Assessment, Hammersmith Infant Neurological Examination (HINE) scores, and results of formal audiology testing at postmenstrual age 52 to 54 weeks. When the MRI, General Movements Assessment, and HINE are all outside reference ranges and indicating cerebral palsy (CP), the sensitivity for detecting CP is 98% with a specificity of 99%.^12^ Formal diagnosis of normal, delayed development, or early features of CP or high risk of CP at that age was also noted.

### Data Acquisition and Storage

Maternal and infant demographics and infant MRI and neurodevelopmental outcome data were obtained from the Monash Health electronic or scanned medical records or from Monash Imaging software, as appropriate. Data were analyzed and securely stored on password-protected Monash Health and Monash University servers and on a CORD-SaFe study–dedicated REDCap database (Vanderbilt), which was accessible only by study investigators (L.Z., A.R., K.C., and A.M.).

### Statistical Analysis

Statistical analyses were conducted using Stata software version 18 (StataCorp) from October to December 2024. Binary variables were expressed as counts and percentages, while continuous outcomes were summarized as medians with IQRs. Comparisons between groups for binary variables were made using the χ^2^ test or Fisher exact test, and continuous variables were analyzed using the Wilcoxon rank-sum test. Regression analyses were performed to adjust for potential confounders that differed significantly between groups. Quantile regression was applied to continuous variables, such as MRI brain injury score and HINE score, while ordinal logistic regression was used to evaluate early neurodevelopmental outcomes, categorized as normal, developmental delay, or CP or high risk of CP, and a logistic regression was performed for absent fidgety movements. *P* values were 2-sided, and statistical significance was set at P ≤ .05.

## Results

The UCBC group included 23 infants (median [IQR] gestation, 26 [25-27] weeks; median [IQR] birth weight, 748 [645-981] grams; 17 [73.9%] male) and the contemporaneous group included 93 infants (median [IQR] gestation, 26 (24-27) weeks; median [IQR] birth weight, 769 [660-1017] grams; 39 [41.9%] male). Infants in the infused group were administered UCBCs at a median (IQR) dose of 42.3 (31.1-63.2) million cells/kg between postnatal days 12 and 14. Among infants in the UCBC group, 15 received the infusion while receiving noninvasive continuous positive airway pressure respiratory support, while 8 were receiving invasive respiratory support (3 receiving conventional mechanical ventilation and 5 receiving high frequency oscillatory ventilation).

Demographic data are presented in Table 1, including maternal and neonatal perinatal data, neonatal data preinfusion, and information on neonatal morbidities during neonatal intensive care unit (NICU) admission, all of which have been previously reported. There was 1 death after enrollment in the UCBC cohort (due to bacterial sepsis, not related to UCBC infusion) and 2 deaths in the contemporaneous cohort.

**Table 1.** Demographic Data for Infants Receiving Autologous UCBC Infusion and a Contemporaneous Cohort Not Receiving UCBC Infusion

Demographics	Infants, No./total No. (%)	
	Cell therapy cohort (n = 23)	Contemporaneous cohort (n = 93)
Perinatal characteristics		
Maternal age, mean (SD), y	35 (30-37)	32 (29-35)
Maternal BMI, median (IQR)	28 (23-32)	27 (24-33)
Pregnancy-induced hypertension	4/23 (17.3)	11/93 (11.8)
Gestational diabetes	7/23 (30.4)	11/93 (11.8)
Chorioamnionitis	6/23 (26.0)	6/93 (6.4)
Prolonged rupture of membranes (>18 h)	12/23 (52.1)	35/93 (37.6)
Antepartum hemorrhage	3/23 (13.0)	27/93 (29.0)
Fetal growth restriction	8/23 (34.7)	16/93 (17.2)
Antenatal steroids (complete course)	22/23 (95.6)	60/93 (64.5)
Magnesium sulfate at delivery (any)	23/23 (100)	79/93 (84.9)
Cesarean delivery	19/23 (82.6)	51/93 (54.8)
Deferred cord clamping (≥60 s)	16/23 (69.5)	66/93 (70.9)
Intubation at birth	6/23 (26.0)	27/93 (29.0)
Neonatal characteristics		
Gestational age at birth, median (IQR), completed wk	26 (25-27)	26 (24-27)
Birth weight, median (IQR), g	750 (650-946)	769 (660-1017)
Sex		
Female	6/23 (26.1)	54/93 (58.1)
Male	17/23 (73.9)	39/93 (41.9)
Apgar score at 5 min, median (IQR)	8 (6-9)	8 (7-8)
IVH grade 1-2	13/22 (59.0)	48/93 (51.6)
Patent ductus arteriosus treated	13/22 (59.0)	42/93 (45.1)
Neonatal morbidities		
Any major morbidity	22/23 (95.6)	79/93 (84.9)
Death	1/23 (4.3)	2/93 (2.1)
Late-onset sepsis	13/22 (59.1)	35/88 (39.7)
NEC requiring surgery	3/22 (13.6)	7/85 (8.2)
ROP requiring treatment	4/22 (18.1)	9/90 (10.0)
BPD at PMA 36 wk	19/22 (86.3)	75/93 (80.6)
Length of stay, median (IQR), d	108 (90-140)	98 (71-135)

Abbreviations: BMI, body mass index (calculated as weight in kilograms divided by height in meters squared); BPD, bronchopulmonary dysplasia; IVH, intraventricular hemorrhage; NEC, necrotising enterocolitis; PMA, postmenstrual age; ROP, retinopathy of prematurity.

### MRI Brain Data

Median (IQR) MRI brain injury score was 2 (1-3) for the UCBC group and 3 (2-5) for the contemporaneous cohort (Table 2). The adjusted analyses revealed no statistically significant difference between the groups (adjusted median difference, 0 [95% CI, −1.78 to 1.78]). For both groups, the global MRI brain injury scores were in the normal (0-3) or mild (4-7) category. Brain structure measurements between groups also were not significantly different (eTable in Supplement 2).

**Table 2.** Comparison of Brain Imaging and Early Neurodevelopmental Outcomes Between Groups

Outcome	Cell therapy cohort (n = 22)	Contemporaneous cohort (n = 93)^a^	Adjusted analysis	
			Effect estimate (95% CI)^b^	*P* value
MRI score, median (IQR)	2 (1 to 3)	3 (2 to 5)	0 (−1.78 to 1.78)	1.00
Early neurodevelopmental assessments, No./No (%)				
Normal	13/22 (59.1)	58/87 (66.6)	0.31 (−0.76 to 1.38)	.56
Delay	9/22 (40.9)	23/87 (26.4)		
Early diagnosis of CP	0/22 (0.0)	6/87 (6.8)		
HINE score, median (IQR)	57 (51 to 61)	59 (55 to 63)	−1.50 (−5.78 to 2.78)	.48
Absent fidgety general movements, No./No (%)	1/22 (4.5)	10/87 (11.5)	0.24 (0.20 to 3.04)	.27
Sensorineural hearing loss	1/18 (5.5)	3/61 (4.9)	NA	

Abbreviations: CP, early features of cerebral palsy or high risk of cerebral palsy; HINE, Hammersmith infant neurological examination; MRI, magnetic resonance imaging; NA, not applicable (regression analysis not performed due to low event rate).

^a^ For the contemporaneous cohort, 77 infants (82.8%) underwent MRI, and 87 neuodevelopment assessments (93.5%).

^b^ Adjusted for sex, antenatal steroids, magnesium sulfate, chorioamnionitis, gestational diabetes, and mode of delivery, with effect estimates representing β for MRI and HINE scores, odds ratio for absent fidgety, and log odds coefficient for early neurodevelopmental diagnosis.

### Early Neurodevelopmental Outcomes

The median (IQR) HINE score was 57 (51-61) in the UCBC group and 59 (55-63) in the contemporaneous group, with no statistically significant difference observed. Absent fidgety general movements was identified in 1 of 22 infants (4.5%) in the UCBC group, compared with 10 of 87 infants (11.5%) in the contemporaneous group, with no statistically significant difference between groups. Lastly, no infants in the UCBC group were assessed as at high risk for CP, while 6 of 87 assessed infants (6.8%) received a high risk of CP diagnosis in the contemporaneous group; however, the difference was not statistically significant (Table 2).

## Discussion

The CORD-SaFe nonrandomized trial established the feasibility of adequate cord blood collection and safety of autologous UCBC administration in extremely preterm infants, addressing a population distinct from previous similar studies that primarily focused on moderately or very preterm infants. In this preplanned follow-up study, early neurodevelopmental outcomes are reported. MRI brain scans at term-equivalent age, and standardized neurodevelopment assessments were completed at postmenstrual age 52 to 54 weeks, with results demonstrating no significant differences between groups. This is in the context of a phase 1, single-group study with a contemporaneous untreated cohort used for comparison. The findings are exploratory, given the study was not randomized or sufficiently powered to find differences in these secondary outcomes.

Additionally, no statistically significant differences in early developmental outcomes were found between groups on regression analysis; however, no infants in the cell infused group were assessed as at high risk for CP, while 6.8% of infants in the contemporaneous received a high risk of CP diagnosis. Given the low occurrence of CP, it is challenging to detect meaningful differences, even in relatively larger studies, and the small sample size of this study further limits the ability to draw definitive conclusions. Nonetheless, these preliminary findings are encouraging, and a larger study has commenced to further investigate the potential benefits of autologous cord blood–derived cell therapy in this cohort.^13^

Study participants and the contemporaneous cohort infants are undergoing ongoing follow-up assessments with standardized developmental assessments at corrected age 2 to 3 years using the Bayley Scales of Infant and Toddler Development–Fourth Edition (Bayley-4). These results will be reported when available.

UCBC therapy is being increasingly evaluated for neonatal conditions,^14^ as it is a rich source of stem and progenitor cells, which have been shown to have beneficial effects on neuropathology related to perinatal brain injury in preclinical models.^6,7^ It is important to note the variations in cell types used in these studies and early phase clinical trials. Some studies have used cord blood–derived mesenchymal cells,^15,16,17^ while others have used cord-blood mononuclear cells,^18^ with potential for differential actions. In our study, we used a mix of cells contained in the buffy coat, known as *total nucleated cells*, which include a diverse population of cells that may collectively contribute to their therapeutic potential. Particularly, our group’s preclinical work has shown that these cells appear to better protect white matter development in the preterm brain in response to inflammation-induced brain injury.^16^ The factors influencing the efficacy of UCBC therapies in perinatal brain injury are still not completely understood, and more preclinical and clinical studies are needed.^19^

Two-year neurodevelopmental assessments have been the traditional primary outcome measures in neonatal intervention trials. However, there is growing use of early neurodevelopmental assessments and MRI to better understand and predict neurological outcomes at an earlier stage.^4,20^ We have reported on the predictive value and accuracy of early neurodevelopmental assessments in predicting 2-year outcomes, and have shown that they are helpful to predict physical or motor outcomes but not cognitive outcomes.^4^ Although we did not observe a statistically significant difference, the absence of any infants with a high risk of CP in the group of infants who received autologous UCBC therapy is promising. This preliminary finding from a phase 1 study warrants further exploration in an adequately powered randomized clinical trial.

### Limitations and Strengths

The study has several limitations, and results must be interpreted with caution. As a single-center, phase 1 safety and feasibility trial, the findings may not be generalizable to other centers and populations. This includes the absence of data on race, as this is not routinely collected in the treating institution. Additionally, the study was not a randomized trial and was not powered to assess the efficacy of autologous UCBC infusion, making it difficult to draw definitive conclusions about its clinical impact. While all infants in the UCBC therapy group completed MRI brain scans and follow-up, there were some missing data in the contemporaneous group, as not all infants underwent MRI imaging and standardized neurodevelopmental testing

Notwithstanding these limitations, this phase 1 clinical trial demonstrated feasibility of collection and safety of infusion of intravenous autologous UCBC therapy in extremely preterm infants,^8^ which is the key strength of the study. Additionally, this report on early neurodevelopmental outcomes is important, as data on infants exposed to autologous UCBC therapy are currently lacking. The use of MRI and the Kidokoro score to assess brain injury is another strength, as this approach is novel, providing valuable insights into early brain development and has not been widely applied in similar studies.

## Conclusions

Early neurodevelopmental outcomes of infants in the CORD-SaFe nonrandomized trial support and justify advancing to the next phase of the study. The CORD-CELL randomized controlled trial^13^ which we are currently undertaking, has the objective to investigate efficacy of autologous UCBCs for long-term neuroprotection in extremely preterm infants.

## Notes

Supplement 1.
Trial Protocol

Supplement 2. **eTable.** Comparison of measurements (in mm) of brain structures at term equivalent age between groups

Supplement 3.
Data Sharing Statement
