# Supplementary material for: Early Neurodevelopment of Extremely Preterm Infants Administered Autologous Cord Blood Cell Therapy: Secondary Analysis of a Nonrandomized Clinical Trial
Source: JAMA Netw Open. 2025 Jul 3;8(7):e2521158. doi: 10.1001/jamanetworkopen.2025.21158 (PMC12232180; doi:10.1001/jamanetworkopen.2025.21158)
Supplement: Supplement 1. — Trial Protocol [file jamanetwopen-e2521158-s001.pdf]

Monash Newborn

Handled with Care

Research Protocol

Title: Autologous CORD blood derived stem cells SAfety and FEasibility study in extreme preterm infants

Short title: CORD-SAFE Study

Type of trial: Phase I clinical trial

Principal Investigator: Dr Atul Malhotra

Associate Investigators: Dr Lindsay Zhou, Professor Graham Jenkin, Professor Suzanne Miller

Clinical Location: Monash Newborn, Monash Children's Hospital, Monash Health, Clayton

Research Laboratory: The Ritchie Centre, Translational Research Facility, Hudson Institute of Medical Research, Monash Medical Centre, Clayton

Cell Processing Facility: Cell Care, Heatherton & Cell Therapies Platform, Hudson Institute of Medical Research

Trial statement: This study will be conducted according to the protocol and will conform to good clinical practice and all applicable regulatory requirements.

Background

More than 3000 very preterm infants (less than 32 weeks' gestation) are born every year in Australia and around 7% of them go on to develop cerebral palsy (CP). Preterm brain injury (mostly significant intraventricular haemorrhage and cystic periventricular leukomalacia) complicates the neonatal course of up to 6% of very preterm infants (especially extremely preterm infants, born before 28 weeks' gestation), contributing as the principal cause of CP in this population (1). Most preterm infants who suffer severe or significant preterm brain injury in the neonatal period have very poor prognosis, with a proportion of them dying during the neonatal period, needing shunt surgery for post haemorrhagic hydrocephalus and, in those who survive, many go on to suffer from adverse long-term neurodevelopment, including CP. Advances in neonatal care have substantially improved survival in very preterm and extremely preterm infants with brain injury, but our aim is to prevent or cure motor disability, specifically CP, at the outset. **No early intervention curative treatment is currently available for preterm brain injury.**

Rationale

Stem cell therapies are increasingly being trialled for neuroprotection and neuroregeneration in young children (2-5), including one current trial in young children with confirmed CP in Australia (ACTRN12616000403437) and further trials proposed (SCUBI Phase 2 Trial). There is now substantial preclinical evidence, mainly from our research group, that administration of umbilical cord blood derived mononuclear cells (UCBCs) in the early neonatal period reduces perinatal brain injury and prevents the progression of neuropathology. UCBCs have been shown to be neuroprotective for the preterm brain when administered in pre-clinical models of hypoxic-ischemic and inflammation-induced preterm brain injury (6-8). More recently, our group has shown that a single dose of UCBC therapy delivered improvement in long-term behavioural outcomes in a rat model of neonatal hypoxic ischemic injury (9). Umbilical cord blood can be easily and safely collected at birth, leading to

a high cell yield which contains a wide variety of stem and progenitor cells, that have been shown to mediate positive benefits on a variety of neurological cells, including glial cells, neurons and cells that maintain the blood brain barrier (BBB) (2, 10-12). These, and other, studies show that the neuroprotective and neuroreparative benefits of early UCBC therapy for the developing brain are likely mediated by their anti-apoptotic, anti-inflammatory, pro-angiogenic, neurogenic, antioxidant, and BBB protective mechanisms (4, 6-8). Most of these studies have used term cord blood-derived UCBCs, but our group has also investigated the neuroprotective/neuroreparative characteristics of preterm UCBCs (6, 13). There is one published clinical study which has been conducted in preterm infants using autologous UCBCs, but the gestation age of included infants in that study was greater than 28 weeks (14).

Proposal

We propose that **in extremely premature infants, i) autologous umbilical cord blood cell (UCBC) collection at birth will be feasible and the ii) intravenous administration of processed cord blood stem cells in the neonatal period will be safe.** This will potentially pave the way for cellular therapy to be rigorously tested to improve neurological outcomes of preterm infants and prevent or ameliorate CP in the long run. To this end, we first intend to develop a phase 1 study of UCBC administration in extremely preterm infants to test feasibility of cell collection, processing and safety of cell administration in this vulnerable group. We will also study characteristics of preterm UCBCs and key biomarkers of brain injury to assist in the design of future, larger efficacy trials.

Aims

The overall project goal is to improve the outcomes of preterm infants with brain injury by prevention/reduction of death and burden of adverse neurodevelopment outcomes, including CP.

The specific aims of this study are to test the feasibility and safety of UCBC collection and autologous intravenous administration in extremely preterm infants during the neonatal period. This includes identification, consent and enrolment of women with threatened preterm delivery, collection of cord blood from **extremely preterm infants** for whom parental consent has been obtained, and intravenous administration of autologous UCBCs to eligible infants **within the second week of life**. Those infants who have had UCBC collected, but who do not meet the criteria for cell processing or cell administration, including insufficient blood volume or cell number, will only have their UCBC characteristics studied for comparative purposes. Safety of UCBC administration will be informed by the close monitoring of infant wellbeing during and after cell administration, and by follow up until 2 years corrected age.

Study Objectives

Primary Objectives

The primary objective of this phase I trial is to evaluate the feasibility of sufficient cord blood collection at birth, and safety of UCBC administration to extremely premature infants within the second week of life.

Secondary Objectives

The secondary objectives of this study are to characterise the properties of UCBCs of extreme premature infants; to investigate the immune response to autologous UCBC administration; and to study the neonatal and long-term outcomes of infants following UCBC administration.

Methods

Design: Phase 1, single centre feasibility and safety study.

Setting: Monash Newborn, Monash Children's Hospital, Monash Health

Ethics: The study will be conducted in compliance with the approved protocol/amendment(s), conditions of Monash Health HREC approval and the NHMRC National Statement on Ethical conduct in Human Research 2007 (updated May 2015).

<u>*Cord blood collection*</u>

Participants: **Extreme preterm infants born before 28 completed weeks of gestation** (up to 27^{+6} weeks).

Exclusion criteria for cord blood collection: Infants born after 28 weeks completed weeks of gestation, known major congenital malformation, positive maternal infectious disease serology.

Consent: Fully informed and written consent will be obtained antenatally from the parents after detailed discussion regarding the 'experimental' nature of this study, and potential benefits/lack of benefits of cord blood collection and/ or UCBC administration. Consent will not be obtained during active labour or after birth. In all cases, written consent will be obtained using a specifically designed Participant Information and Consent Form (PICF). A copy of the PICF will be provided to parents, documented in the infant's hospital record and kept in a secure study folder. A Cell Care agreement to collect and store processed cord blood (in accordance with TGA requirement) will also be obtained separately by Cell Care staff.

Cord blood collection and processing: Umbilical cord blood will be collected from all eligible extreme preterm infants (as per participant age above) of parents who have consented to the trial. Size appropriate needles and collection containers/ bags will be used to maximise the volume of cord blood that can be obtained. Minimum cord blood collection volume for inclusion in the trial will be 7 mLs. Infants with collected cord volume of less than 7 mLs will

be excluded from the study, but their cord blood will be retained and its characteristics investigated as part of the comparative cell characterisation study. The collected cord blood (>7 mLs) will be processed according to Standard operating procedures (as in attached documents), aliquoted and cryopreserved.

Storage and release: An aliquot containing sufficient numbers of UCBCs, according to weight of the neonate, required for early administration (see dose below) will be stored. Any excess UCBCs will compose further aliquot(s) that will be stored for potential subsequent use, not as part of the current trial. It is expected that such aliquots could be made available for unexpanded or expanded UCBC use in subsequent trials (not part of this ethics approval). Cell Care (Heatherton, VIC), a TGA-licensed cord blood bank, will be responsible for collection, processing, storage of all samples and release of UCBC for the study. Standard testing for maternal serology, microbial contamination, viable total nucleated cell count, and ABO, Rh, and HLA matching of UCBC to the preterm infant's HLA (to confirm it is autologous) will be performed before administration of UCBCs to the eligible infants. Standard operating procedures for cell collection, processing, storage and release will be followed as detailed in the attached documents. Cell Care has agreed to undertake these services "Pro-Bono".

UCBC administration

Inclusion criteria: **Extreme preterm infants with absence of severe brain injury** on neonatal cranial ultrasound, performed in the first week of life.

Exclusion criteria for UCBC administration: Extreme preterm infants with evidence of severe preterm brain injury as defined by Grade III-IV intraventricular haemorrhage and/ or cystic periventricular leukomalacia. Infants who are likely to have redirection of intensive care due to any reason, as decided by the treating clinical team would be excluded. Any infant who

has a microbiological growth or positive serology on cord blood collection (namely HIV, Hepatitis B/C) will also be excluded.

Intervention: UCBCs from autologous cord blood collection administered intravenously to infants, ideally between **D9 – D15 of life**. This will follow the routine D8 cranial ultrasound scan. Infants with an active bacterial infection (blood culture positive in last 48 hours) or instability as determined by treating team will have cell administration deferred if possible within the treatment window (D9-15 of life). A (heel-prick) blood sample will be taken from eligible infants before UCBC administration, to match the HLAs and confirm the UCBCs are from the baby's own cord blood prior to infusion.

Dose and administration: UCBCs will be administered intravenously (through a peripheral intravenous catheter) at a dose of **25-50 million viable cells/ kg body weight**, 25 million/kg being the proposed minimum dose, and 50 million/kg being the maximum dose. The dose will depend on the number of UCBCs able to be processed and deemed viable after cord blood collection. This dose is based on preclinical (6, 7, 13, 15) and clinical studies (2, 14) on UCBC use for perinatal brain injury. If the number of cells available is less than 25 million/kg, then cell administration will not occur in this trial but cell characteristics will be studied. Volume of cell infusion will be approximately 10 mL/kg and cell infusion will occur over one hour. After release from Cell Care, cells will be transported at Monash Health Translation Precinct laboratories. After thawing, washing of cells will be performed using Dextran-Albumin solution and re-suspended in an appropriate volume of Dextran-Albumin solution. Cell viability will be determined before administration. Cell administration will be based on an and routine cord blood transfusion protocols in children and an ongoing placental stem cell study at Monash Newborn (16). Please see attached Standard operating procedures for detailed description of cell thawing, washing and administration.

Primary outcomes: The primary focus of this study is to test i) feasibility of autologous cord blood collection and cell retrieval following processing extremely preterm infants, and ii) safety of intravenous cell administration in extremely preterm infants.

Feasibility will be determined by agreement to participate, ability to collect and process sufficient cord blood, and then appropriate access to sufficient UCBCs within the second week of life. UCBCs will be collected from all potentially eligible infants but will only be administered to eligible infants as per inclusion and exclusion criteria above. Unused and remaining cells will be stored for potential future use.

Safety of UCBC administration will be determined by occurrence (absence) of adverse events as defined below. Infants will be monitored as follows:

Monitoring during UCBC infusion and nursery stay

Infants will be observed for 2 hours prior to UCBC infusion to determine their baseline cardiorespiratory status and establish acceptable parameters for fluctuations during the infusion. During the infusion, continuous HR, RR, ECG, SpO_2 will be monitored while BP, temperature, and site of infusion will be checked every 15 minutes. Post infusion, continuous HR, RR, ECG, SpO2 will be monitored, while BP, and temperature will be checked hourly.

Infants, as a result of their prematurity, are likely to remain inpatients in neonatal intensive and special care nurseries for at least 3-4 months following the UCBC administration. The routine clinical care afforded infants over this time will serve as monitoring for adverse events.

Routine care will include continuous cardiorespiratory monitoring; physical examination (daily while infants remains on respiratory support); regular anthropometry (at least weekly weight, head circumference, length); documentation of respiratory support requirements;

chest radiograph, as clinically indicated; blood gas analysis, as determined by clinical team; cranial ultrasound, as per clinical practice but a minimum of two cranial ultrasounds post infusion prior to discharge (D28, 42); term equivalent age MRI brain, as per clinical practice.

Monitoring post discharge

Following discharge from nursery, infants will be assessed at 6, 12, 18 and 24 months corrected age. Assessment will focus on general health including growth parameters and physical examination, reporting of any adverse events and medication use.

Neurodevelopmental assessment will be performed using standardised assessment tools, including Prechtl's General Movements Assessment and Hammersmith Infant Neurological Examination (at 3 months corrected age), and the Bayley Scales of Infants and Toddler Development (IV edition) at 2 years corrected age. These are all routine for extreme premature infants at Monash Newborn.

Defining Adverse Events

Adverse events will be defined as follows:

- During infusion
  - Local Site Reaction (Erythema, oedema, extravasation at site of peripheral intravenous catheter site)
  - Any sustained change of 30% or more from baseline in vital signs (HR, RR, BP, SpO2, Temp)
- Within first 24 hours of infusion
  - Any event requiring cardiopulmonary resuscitation
  - Escalation of respiratory support (intubation of an infant receiving non-invasive respiratory support; or change to high frequency oscillatory

ventilation in an infant receiving conventional ventilation at the time of UCBC infusion)
    - Fluid bolus or initiation/escalation of inotropic support
- Infection within 48 hours of UCBC infusion (culture proven bacterial, fungal or viral infection, or culture negative, clinically suspected infection)

Secondary outcomes: Neonatal and long term general health and neurological outcomes, including cranial ultrasound changes after D8 of life, term equivalent MRI brain findings (if clinically indicated), early neurodevelopment assessments in the first few months of age (including General Movements, Hammersmith Infant Neurological Examinations at 3 months corrected age as part of their routine Early Neurodevelopment Clinic), and 2 year neurodevelopment assessment (BSID-IV as part of their routine Growth and Developmental follow-up). A six-monthly (until 2 years of corrected age) clinical examination and documentation of any adverse events in the intervening period will also be conducted by the study principal investigator. A detailed case record form (CRF) is included in the supporting documents. The secondary outcomes of the study participants may also be compared to a matched historical cohort (same criteria of no evidence of severe brain injury by D8 scan) of extremely premature infants.

In a sub study, when sufficient volume of cord blood is not available for infusion (< 7 mL of cord blood), we will characterise the composition, characteristics and functionality of the preterm UCBCs, given there is limited literature on the characteristics of preterm UCBCs. Comparative studies with stored term UCBCs collected for other studies may be considered.

We will also conduct a targeted cytokine analysis on infant's serum; namely IL-1b, IL-6, TNF-α and IL-10 at time (just before) of UCBC administration, 1 day post administration and at 36 -37 weeks postconceptional age as potential biomarkers for response to cell administration.

A 0.2 mL sample of venous blood will be collected in an EDTA or citrate tube and transported to the Laboratory on ice by trial investigators. The samples will be centrifuged and the plasma separated for storage and subsequent analysis in the Ritchie Centre laboratories at Monash Health Translational Research Precinct, Monash Medical Centre Clayton.

Sample size: We aim to administer UCBCs to at least 20 extremely preterm infants. We will further aim to administer cells to at least 10 infants who will receive 50 million/kg UCBCs within the cohort of babies to be studied. So, the total number of infants who may receive UCBCs may be between 20-25.

Withdrawal: Parents may withdraw consent, for any reason, at any time after inclusion in the trial. The reason for withdrawal will be documented in the CRF. Permission will be sought from parents who withdraw consent to allow for continued data collection from the infant's medical record. Should a patient withdraw following the UCBC infusion, they will be offered the six-monthly follow-up designed to monitor for adverse effects. Additional infants will be recruited should participants withdraw to ensure at least 20 infants complete the study till 2 years of age.

Safety Reporting

Categorising safety related events: Events will be categorised as per the National Health and Medical Research Council 2016 recommendation

Adverse event (AE) Any untoward medical occurrence in a patient or clinical trial participant administered a medicinal product and that does not necessarily have a causal relationship with this treatment.

Adverse Reaction (AR) Any untoward and unintended response to an investigational medicinal product related to any dose administered.

Serious AE/AR (SAE/SAR) Any adverse event/adverse reaction that results in death, is life-threatening, requires hospitalisation or prolongation of existing hospitalisation, results in persistent or significant disability or incapacity.

Suspected Unexpected Serious Adverse Reaction (SUSAR) An adverse reaction that is both serious and unexpected.

Data Safety Monitoring Board (DSMB): An independent DSMB has been formed comprising of a neonatologist, and a cell therapist/ biologist to review all AE/ AR/ SAE/ SAR/ SUSARs (See details of DSMB in Appendix below). Adverse incidents will be reported to DSMB within 1 week of occurrence. An interim review will also take place after recruitment of 10 babies.

Reporting to HREC: The following reports will be submitted to HREC:

1. Any adverse event/ reaction.
2. An interim report after recruitment of 10 babies.
3. Annual research progress report.
4. Any updates to protocol/ PICF.

Data management

Data Collection and Storage: Data will be collected prospectively by trial investigators and sourced from the infant and mother's medical records, bedside clinical charts, electronic medical records. Data will be collected on a paper CRF and recorded in an electronic database. Paper records will be stored in a locked filing cabinet and electronic records will be password protected.

Retention of Data: Data will be securely retained for 25 years.

Statistical Methods: As this is a phase I trial, detailed statistical analysis will not be needed, but descriptive and inferential statistical analysis will be conducted as appropriate. Comparative analysis will be performed between study participants and historical controls.

Expected outcomes

We anticipate that early administration of autologous UCBC to extremely preterm infants will be feasible and safe at a dose of 25-50 million cells/ kg.

Around 100 preterm infants born less than 28 weeks' gestation are admitted to Monash Newborn each year. We anticipate to complete recruitment within 12 months of commencement.

The information obtained from this study will enable us to design the next phase of our studies; a randomised controlled trial of efficacy of early administration of autologous UCB to extremely preterm infants. Data may be used to support a NHMRC clinical trial and cohort studies application in 2022.

Participant flow

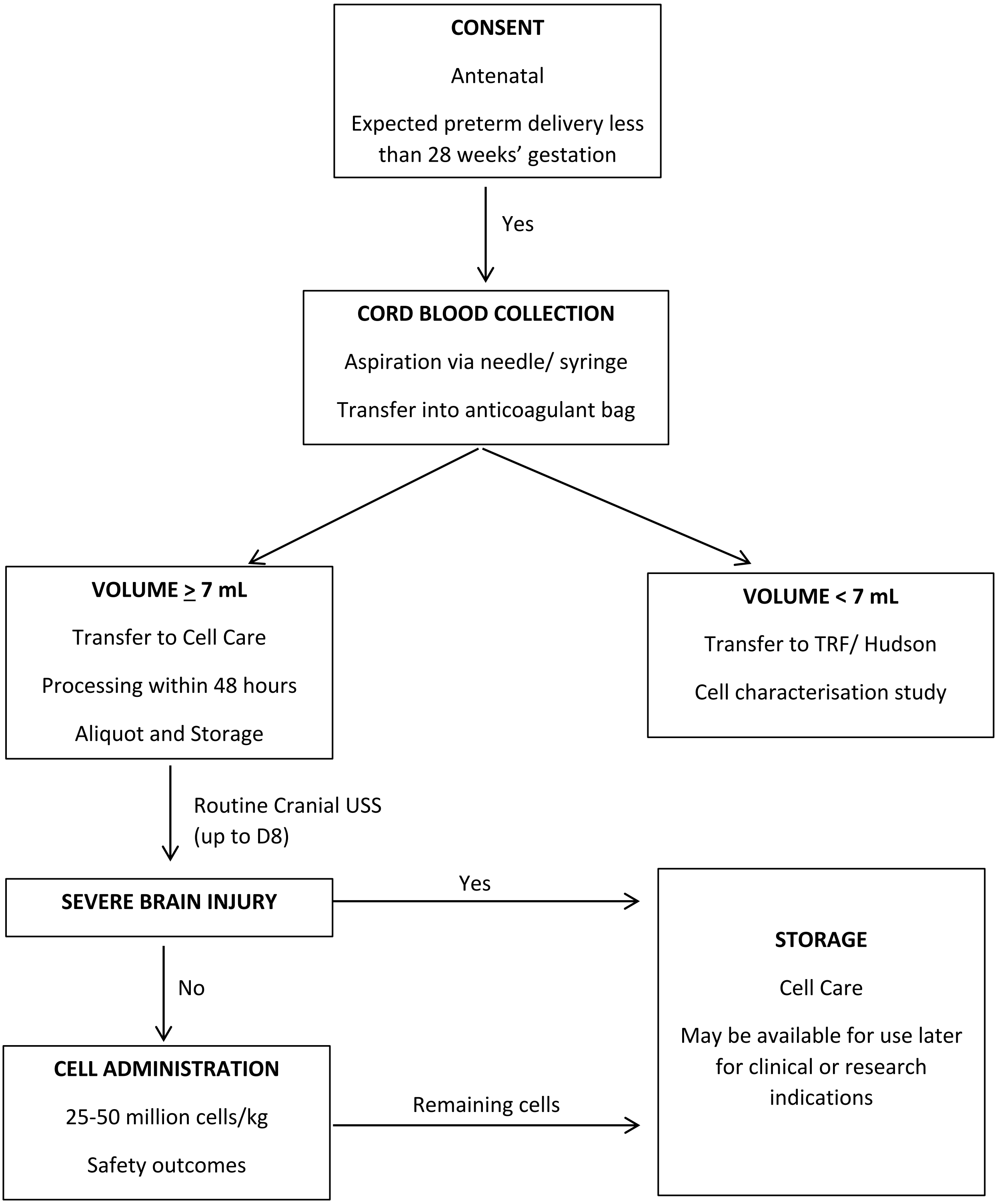

References

1. Chow SSW, Creighton, P., Kander, V., Haslam, R., Lui, K. Report of the Australian and New Zealand Neonatal Network 2016 Sydney: ANZNN; 2018 [Available from: https://www.anznn.net/Portals/0/AnnualReports/Report of the Australian and New Zealand Neonatal Network 2016.pdf.
2. Cotten CM, Murtha AP, Goldberg RN, Grotegut CA, Smith PB, Goldstein RF, et al. Feasibility of autologous cord blood cells for infants with hypoxic-ischemic encephalopathy. J Pediatr. 2014;164(5):973-9.e1.
3. Hattori T, Sato Y, Kondo T, Ichinohashi Y, Sugiyama Y, Yamamoto M, et al. Administration of umbilical cord blood cells transiently decreased hypoxic-ischemic brain injury in neonatal rats. Developmental neuroscience. 2015;37(2):95-104.
4. Aridas JD, McDonald CA, Paton MC, Yawno T, Sutherland AE, Nitsos I, et al. Cord blood mononuclear cells prevent neuronal apoptosis in response to perinatal asphyxia in the newborn lamb. J Physiol. 2016;594(5):1421-35.
5. Min K, Song J, Kang JY, Ko J, Ryu JS, Kang MS, et al. Umbilical cord blood therapy potentiated with erythropoietin for children with cerebral palsy: a double-blind, randomized, placebo-controlled trial. Stem cells (Dayton, Ohio). 2013;31(3):581-91.
6. Li J, Yawno T, Sutherland A, Loose J, Nitsos I, Allison BJ, et al. Term versus preterm cord blood cells for the prevention of preterm brain injury. Pediatr Res. 2017.
7. Li J, Yawno T, Sutherland A, Loose J, Nitsos I, Bischof R, et al. Preterm white matter brain injury is prevented by early administration of umbilical cord blood cells. Experimental neurology. 2016;283(Pt A):179-87.
8. Paton MCB, Allison BJ, Li J, Fahey MC, Sutherland AE, Nitsos I, et al. Human Umbilical Cord Blood Therapy Protects Cerebral White Matter from Systemic LPS Exposure in Preterm Fetal Sheep. Developmental neuroscience. 2018;40(3):258-70.
9. McDonald CA, Penny TR, Paton MCB, Sutherland AE, Nekkanti L, Yawno T, et al. Effects of umbilical cord blood cells, and subtypes, to reduce neuroinflammation following perinatal hypoxic-ischemic brain injury. Journal of neuroinflammation. 2018;15(1):47.
10. Phillips AW, Johnston MV, Fatemi A. The potential for cell-based therapy in perinatal brain injuries. Translational stroke research. 2013;4(2):137-48.
11. McDonald CA, Fahey MC, Jenkin G, Miller SL. Umbilical cord blood cells for treatment of cerebral palsy; timing and treatment options. Pediatr Res. 2017.
12. Jantzie LL, Scafidi J, Robinson S. Stem cells and cell-based therapies for cerebral palsy: A call for rigor. Pediatr Res. 2017.
13. Li J, Yawno T, Sutherland AE, Gurung S, Paton M, McDonald C, et al. Preterm umbilical cord blood derived mesenchymal stem/stromal cells protect preterm white matter brain development against hypoxia-ischemia. Experimental neurology. 2018;308:120-31.
14. Yang J, Ren Z, Zhang C, Rao Y, Zhong J, Wang Z, et al. Safety of Autologous Cord Blood Cells for Preterms: A Descriptive Study. Stem cells international. 2018;2018:5268057.
15. Paton MCB, Allison BJ, Fahey MC, Li J, Sutherland AE, Pham Y, et al. Umbilical cord blood versus mesenchymal stem cells for inflammation-induced preterm brain injury in fetal sheep. Pediatr Res. 2019.
16. Baker EK, Malhotra A, Lim R, Jacobs SE, Hooper SB, Davis PG, et al. Human amnion cells for the prevention of bronchopulmonary dysplasia: a protocol for a phase I dose escalation study. BMJ Open. 2019;9(2):e026265.

Appendix

Details of Independent DSMB

Professor Jeanie Cheong (Neonatologist)
Neonatal Paediatrician, Department of Neonatal Services
Principal Research Fellow, Co-Group Leader Victorian Infant Brain Studies, Murdoch Children's Research Institute
Professor, University of Melbourne
The Royal Women's Hospital, Locked Bag 300, Cnr Grattan St & Flemington Rd, Parkville VIC 3052
P: +61 3 8345 3771 | F: +61 3 8345 3789
E: jeanie.cheong@thewomens.org.au

Professor Richard Boyd (Stem Cell Biologist)
Professor, Immunology, Monash University
Chief Scientific Officer, Cartherics
Co-Director Australia – China Centre for Excellence in Stem Cells
Distinguished Scientist, Hudson Institute of Medical Research
E: richard.boyd@hudson.org.au
