# Supplementary material for: Early Neurodevelopment of Extremely Preterm Infants Administered Autologous Cord Blood Cell Therapy: Secondary Analysis of a Nonrandomized Clinical Trial
Source: JAMA Netw Open. 2025 Jul 3;8(7):e2521158. doi: 10.1001/jamanetworkopen.2025.21158 (PMC12232180; doi:10.1001/jamanetworkopen.2025.21158)
Supplement: Supplement 2. — eTable. Comparison of measurements (in mm) of brain structures at term equivalent age between groups [file jamanetwopen-e2521158-s002.pdf]

Supplemental Online Content

Zhou L, Razak A, McDonald CA, et al. Early neurodevelopment of extremely preterm infants administered autologous cord blood cell therapy: a secondary analysis of a nonrandomized clinical trial. *JAMA Netw Open*. 2025;8(7):e2521158.
doi:10.1001/jamanetworkopen.2025.21158

eTable. Comparison of measurements (in mm) of brain structures at term equivalent age between groups

This supplemental material has been provided by the authors to give readers additional information about their work.

© 2025 Zhou L et al. *JAMA Network Open*.

Supplementary eTable1: Comparison of measurements (in mm) of brain structures at term equivalent age between groups.

	**Cell therapy Cohort (N=23)**	**Contemporaneous cohort (N=77)**
BFD (median (IQR))	60.5 (57, 64.2)	58.5 (54.1, 63.5)
L FLH (mean (SD))	40.7 (6.6)	38.7 (8.3)
R FLH (median (IQR))	42.7 (34.4, 44.8)	39.4 (34.6, 45.3)
Brain BPD (median (IQR))	78.1 (75, 81.3)	76.1 (73.8, 80.8)
Bone BPD (median (IQR))	83.3 (78, 88.1)	79.9 (77.5, 83.9)
TCD (median (IQR))	50.3 (48.3, 50.3)	48.9 (46.9, 52.2)
FOD (median (IQR))	96.6 (94.8, 101.2)	97.4 (92.6, 103.5)
CC length (median (IQR))	39.2 (35.9, 41.1)	39 (36.8, 42.2)
Vermis (median (IQR))	16.4 (14.2, 19.6)	17.9 (16.2, 19.8)
EAS CCIPD L (median (IQR))	2.1 (1.6, 3.1)	2 (1.5, 2.5)
EAS CCIPD R (median (IQR))	2 (1.8, 2.5)	2.1 (1.7, 2.7)
EAS IHD (median (IQR))	4 (3.5, 5)	3.8 (2.7, 5.2)
ICS Third Ventricle (median (IQR))	3.3 (2.7, 4.1)	3.3 (2.5, 4)
R LVAD (median (IQR))	5.4 (4.5, 6.3)	5.8 (5.1, 6.7)
L LVAD (median (IQR))	5.9 (5.2, 6.4)	5.9 (5.1, 7.3)

Note: BFD: bifrontal diameter, FLH: frontal lobe height, BPD: biparietal diameter, TCD: transcerebellar diameter, FOD: fronto-occipital distance, CC: corpus callosum length, Vermis: measure of cerebellar vermis in midsagittal plane, EAS: extra-axial space, CCIPD: craniocaudal inter-opercular distance, IHD: interhemispheric distance, ICS: intracerebral space, LVAD: lateral ventricular atrial diameter

© 2025 Zhou L et al. *JAMA Network Open*.

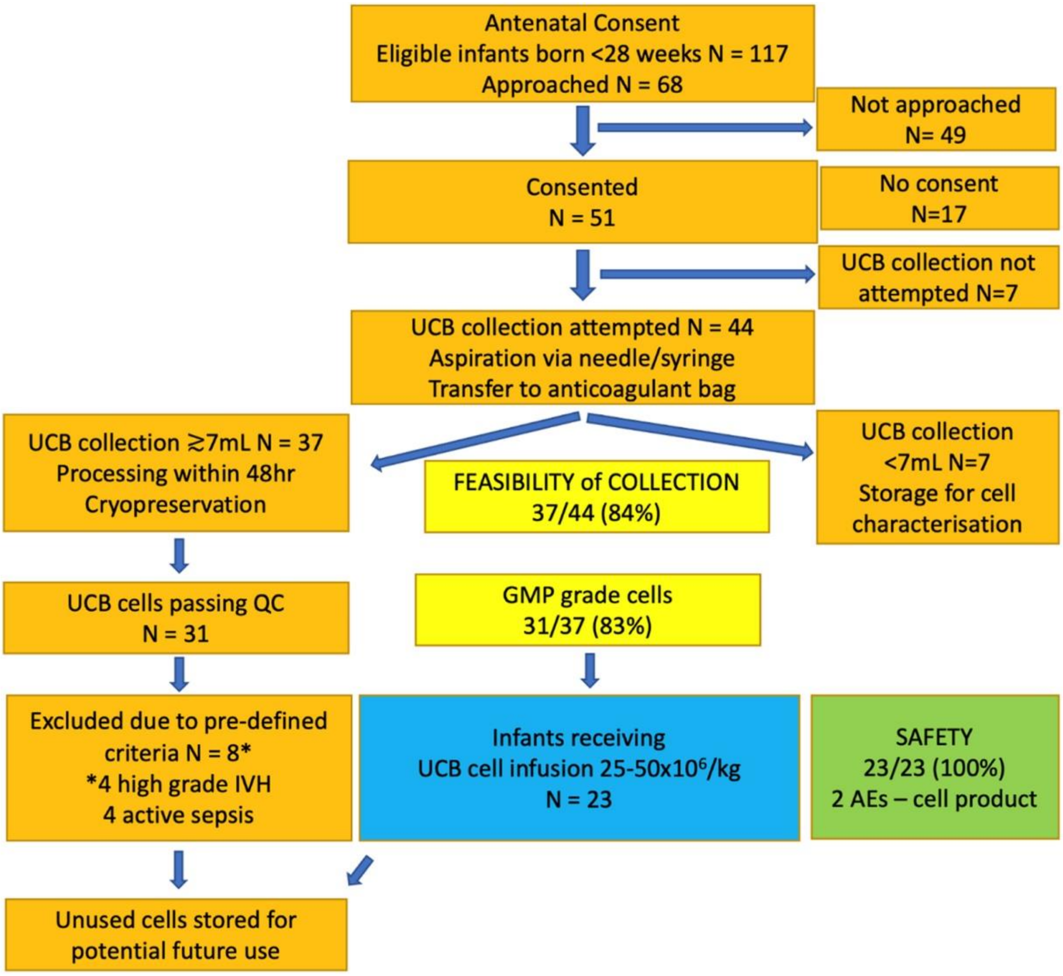

© 2025 Zhou L et al. *JAMA Network Open*.
