# Supplementary material for: Early Neurodevelopment of Extremely Preterm Infants Administered Autologous Cord Blood Cell Therapy: Secondary Analysis of a Nonrandomized Clinical Trial
Source: JAMA Netw Open. 2025 Jul 3;8(7):e2521158. doi: 10.1001/jamanetworkopen.2025.21158 (PMC12232180; doi:10.1001/jamanetworkopen.2025.21158)
Supplement: Supplement 3. — Data Sharing Statement [file jamanetwopen-e2521158-s003.pdf]

Data Sharing Statement

Zhou. Early Neurodevelopment of Extremely Preterm Infants Administered Autologous Cord Blood Cell Therapy. *JAMA Netw Open*. Published July 14, 2025.
doi:10.1001/jamanetworkopen.2025.21158

Data
Additional Information: Clinical Trial Registration: The study was prospectively registered with the Australian New Zealand Clinical Trials Registry: ACTRN12619001637134.
Data available: Yes
Data types: Deidentified participant data
How to access data: Data available on request
When available: With publication

Supporting Documents
Document types: None

Additional Information
Who can access the data: On request
Types of analyses: For systematic reviews
Mechanisms of data availability: Through corresponding author on reasonable request
